# The change of conditions does not affect Ros87 downhill folding mechanism

**DOI:** 10.1038/s41598-020-78008-8

**Published:** 2020-12-03

**Authors:** Rinaldo Grazioso, Sara García-Viñuales, Gianluca D’Abrosca, Ilaria Baglivo, Paolo Vincenzo Pedone, Danilo Milardi, Roberto Fattorusso, Carla Isernia, Luigi Russo, Gaetano Malgieri

**Affiliations:** 1 Department of Environmental, Biological and Pharmaceutical Sciences and Technologies, Via Vivaldi 43, 81100 Caserta, Italy; 2 Institute of Crystallography-CNR, Via Paolo Gaifami 18, 95126 Catania, Italy

**Keywords:** Bioinorganic chemistry, Metals, Protein folding, Proteins, Structural biology

## Abstract

Downhill folding has been defined as a unique thermodynamic process involving a conformations ensemble that progressively loses structure with the decrease of protein stability. Downhill folders are estimated to be rather rare in nature as they miss an energetically substantial folding barrier that can protect against aggregation and proteolysis. We have previously demonstrated that the prokaryotic zinc finger protein Ros87 shows a bipartite folding/unfolding process in which a metal binding intermediate converts to the native structure through a delicate barrier-less downhill transition. Significant variation in folding scenarios can be detected within protein families with high sequence identity and very similar folds and for the same sequence by varying conditions. For this reason, we here show, by means of DSC, CD and NMR, that also in different pH and ionic strength conditions Ros87 retains its partly downhill folding scenario demonstrating that, at least in metallo-proteins, the downhill mechanism can be found under a much wider range of conditions and coupled to other different transitions. We also show that mutations of Ros87 zinc coordination sphere produces a different folding scenario demonstrating that the organization of the metal ion core is determinant in the folding process of this family of proteins.

## Introduction

Protein folding is essentially an exploration of conformational space on a funneled energy surface. Several small protein domains fold by two-state equilibrium mechanisms^1^. Larger domains frequently populate, both transitorily or at equilibrium, intermediate conformations^2,3^ that can be on-pathway or off-pathway. In this latter case, the intermediate conformation may act as structural traps leading to aggregation phenomena^4,5^.

Metal-binding proteins represent more than 30% of all folded proteins^6^. In these proteins the metal cofactor either plays a simply structural role (the most common example is zinc in zinc-finger containing proteins^7,8^) or plays enzymatic roles (i.e., zinc in metallo-proteases^9^) to obtain the functional protein. It is also well-known that several proteins involved in degenerative disorders have metal-binding abilities^10–13^. Metal-binding happens in cellular environments where the proper metal is either present free in solution or bound to delivery proteins^14^. However, many in vitro studies have proved that the metal cofactor has clear influences on the structure, stability and conformational dynamics of its cognate protein^5,15^. Much experimental and theoretical effort has been dedicated to the study of peptides^16–18^ and proteins^19,20^ folding mechanisms but the comparison between different studies can be limited by differences in the protein constructs and by a variety of experimental conditions and methods employed^21^.

**Figure 1:**
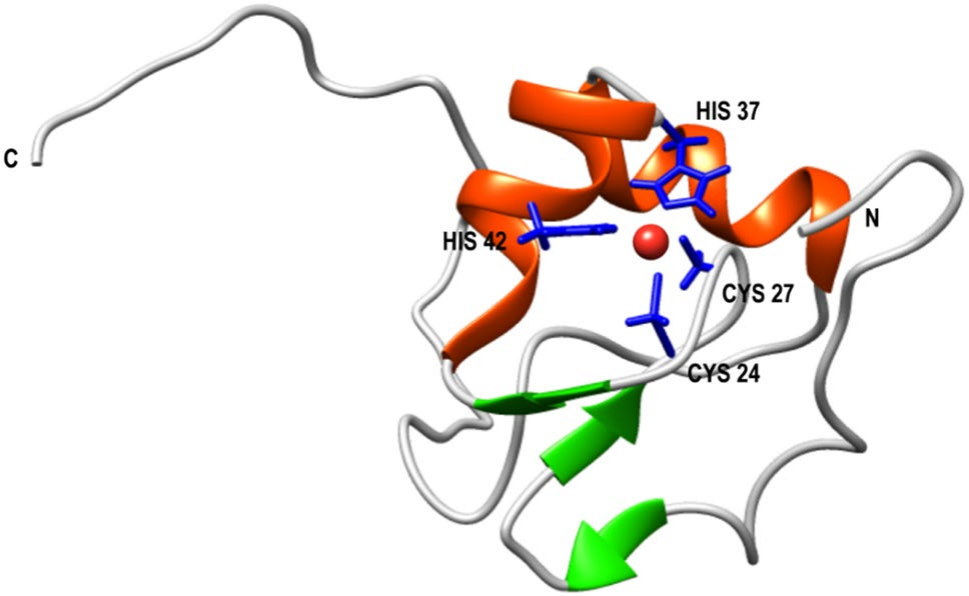
Representative NMR structure of Ros87 (PDB code: 2JSP). α-helices are colored in red and β-strands in green. Side-chains of metal coordinating residues are in magenta.

Our group has lately carried out the characterization of the folding mechanism of a metallo-protein belonging to the prokaryotic zinc finger family that we have named Ros87^5,15^ (Fig. 1).

This protein, deletion mutant of Ros from *A. tumefaciens*^22^, shares with the eukaryotic domain the tetrahedral coordination of Zn(II) by means of two cysteines and two histidines but differs in the secondary structures topology (Ros87 has a βββαα topology) and in the formation of a large hydrophobic core made by 15 residues^23^. The study of different members of this protein family^24–26^ allowed us to demonstrate that this protein architecture to functionally fold can substitute the structural ion with a different set of amino acids or can even surrogate the stabilizing role of the metal cofactor with a network of H-bonds and hydrophobic interactions^25,27–29^. These differences among iso-structural proteins led to different mechanisms of folding: the metal lacking Ml4_52–151_ folds/unfolds with a classical two state mechanism while the coordination of the structural zinc confers to Ros87 a partly down-hill unfolding mechanism. As matter of fact, Ros87 shows a bipartite folding/unfolding process in which a metal binding intermediate converts to the native structure through a delicate barrier-less transition. Downhill folders are expected to be quite rare in nature as they miss an energetically significant folding barrier that can protect against aggregation and proteolysis^30^. Muñoz and co-workers have characterized the downhill folding pathway of the BBL domain^31–34^ as a unique thermodynamic state entailing a conformations ensemble that progressively loses structure with the decrease of protein stability^35^. BBL stability appears particularly sensitive to construct length, the existence of extrinsic fluorophores, ionic strength and pH^30,36^.

Considering that important variation in folding mechanism can be observed within families of proteins with very similar folds and high sequence identity^37^ and for the same sequence by varying conditions^38^, we here describe the characterization of the unfolding mechanism of Ros87 and of one of its mutants in a different buffer with different pH and ionic strength conditions. We have chosen the Tris buffer, a buffer widely used in the characterization of the structure and folding of many proteins. We demonstrate that also in different conditions Ros87 retains the partly downhill folding mechanism demonstrating how the scenario described in the previous work is not simply due to a uniqueness of a particular set of experimental conditions.

## Results

### Ros87 thermal unfolding

**Figure 2:**
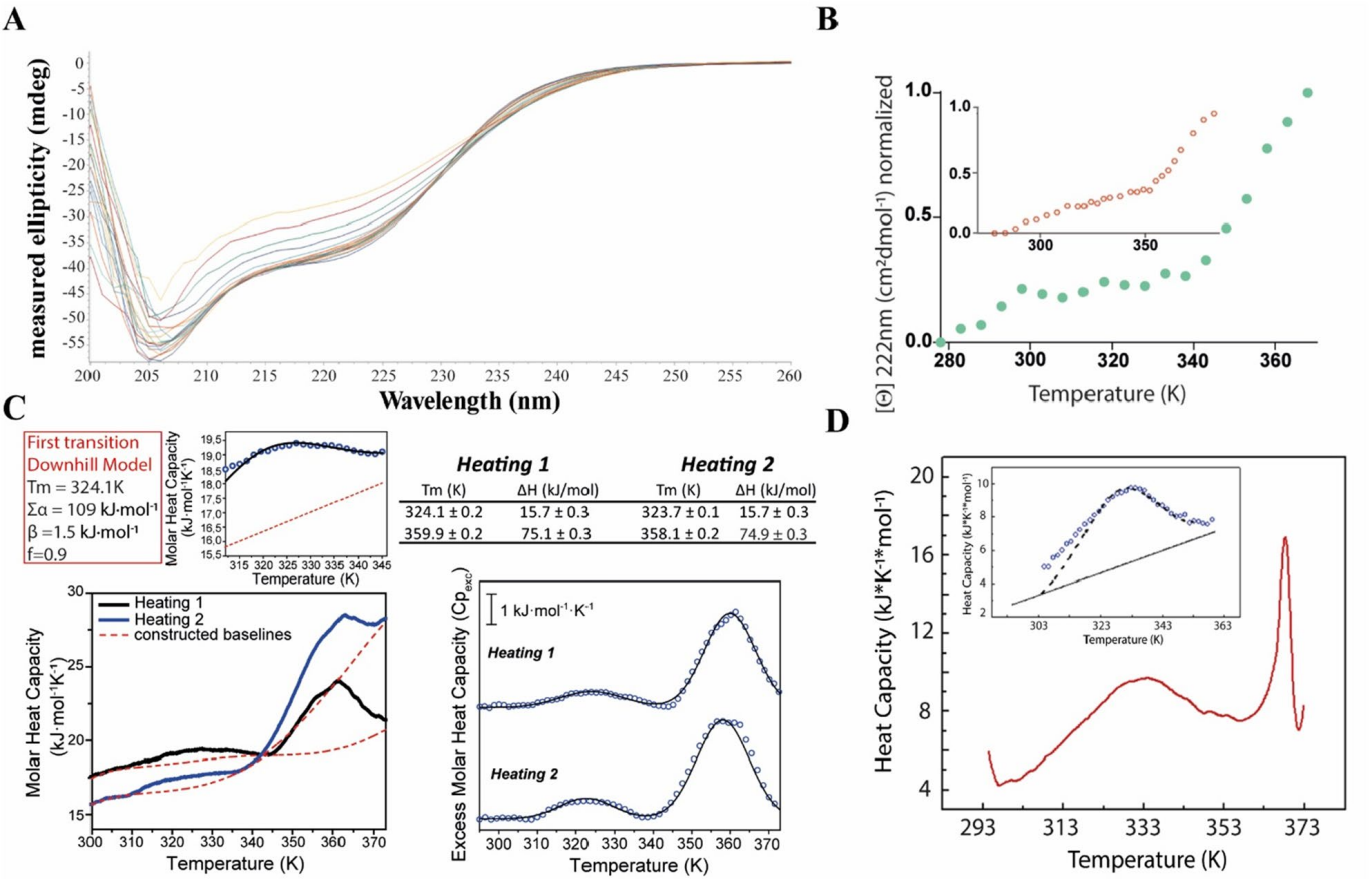
*Ros87 thermal unfolding.* (**A**) CD spectra recorded in Tris buffer at pH = 6.5 in the temperature range 298 K-372 K. (**B**) CD melting in Tris buffer followed at 222 nm in the temperature range 298–372 K; the inset shows the melting followed at 222 nm in phosphate buffer at pH = 6.5^15^. (**C**) DSC thermal unfolding curves and fitting of two different heating cycles in Tris buffer. Baselines are reported with a dashed line. (**D**) DSC thermal unfolding curves and fitting in phosphate buffer^15^. Panel C and D were adapted from Ref.^15^.

The investigation of Ros87 unfolding mechanism started with the analysis of its thermal denaturation via far-UV CD spectroscopy in 10 mM Tris buffer, 150 µM TCEP, pH = 6.5 (Fig. 2A). The protein shows a complex reversible unfolding pathway spanning a temperature range of 83 K, between 295 and 372 K, that cannot be fitted using a single cooperative two-state model (Fig. 2A,B and Figure SI 1). Accordingly, the DSC thermogram shows a first broad, reversible endotherm centered at about 324 K followed by a second also reversible broad endotherm centered at 360 K (Fig. 2C) pinpointing a two steps unfolding process separated by a detectable partially folded intermediate state.

In the previously published DSC thermogram of the same protein in phosphate buffer^15^ (Fig. 2D) this second transition occurs between 370 and 390 K, is quite sharper and irreversible. At first, we have focused our attention on the first transition that in phosphate buffer resulted to be properly described by a downhill scenario. Similar to the former study, also in the new experimental conditions it was not possible to extrapolate from the thermogram the native baseline of this transition. Initially, we fitted the curve by the classic two-state formalism but van’t Hoff to calorimetric enthalpy ratio (ΔH_vH_∕ΔH_cal_) resulted far from 1, supporting the above mentioned CD findings which indicate that the two-state model is inadequate to describe Ros87 thermal unfolding. This prompted us to fit the DSC thermogram to a variable-barrier energy model proposed by Muñoz and Sanchez-Ruiz^33^ obtaining the following parameters: Σα = 109 kJ mol^−1^, T_0_ = 324.1 K, and β = 1.5 kJ mol^−1^ that clearly indicate a downhill scenario. Moreover, this probability distribution is unimodal at all temperatures and varies as normally happens for a one-state transition: with the maximum probability shifts from low-enthalpy values at low temperature to high-enthalpy values at high temperature. Downhill folding for Ros87 has been also confirmed by observing that heat capacity baselines for the native and unfolded protein cross at intermediate temperatures.

We then examined Ros87 thermal unfolding by monitoring temperature-induced chemical shift perturbations using NMR spectroscopy. To this aim, a series of ^1^H–^15^N HSQC spectra were analyzed in a temperature range from 298 to 343 K at regular intervals of 5 K (Figure SI 2). Resonances exhibited a continuous chemical shift variation indicating a fast protein folding process in the µs-ms time scale^39,40^ with most of the residues showing a single sigmoidal behavior and only an exiguous number with a more complex behavior (Figure SI 3).

**Figure 3:**
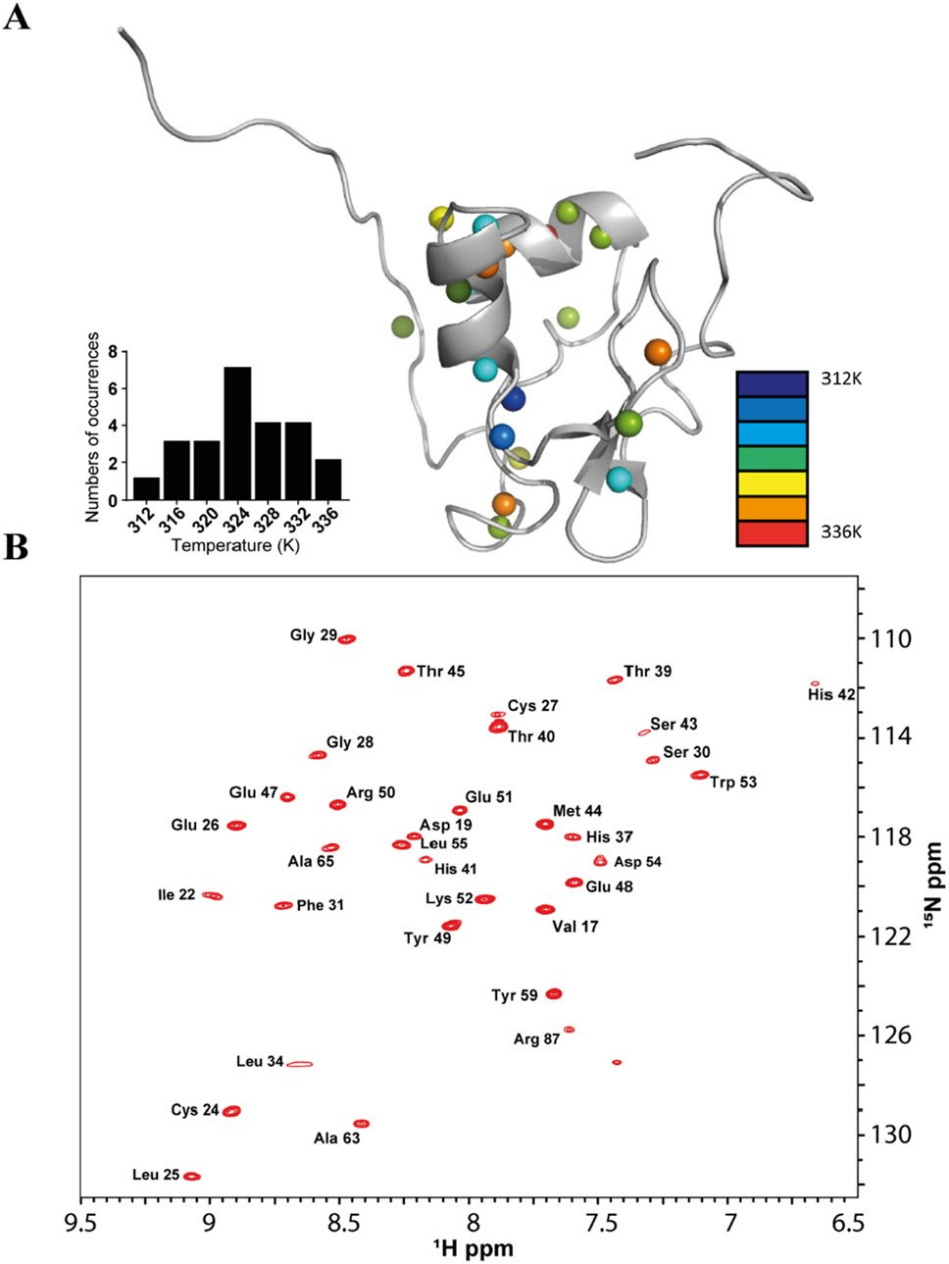
*Ros87 NMR unfolding.* (**A**) “Atom-by-atom” unfolding behavior of Ros87 in 278–343 K range. Ros87 ribbon drawing showing the T_m_ of 24 protons mapped on their corresponding atoms. The inset shows the T_m_ scale. Each atom color corresponds to the T_m_ of its sigmoidal transition. (**B**) ^1^H–^15^N HSQC spectrum at 343 K.

We obtained different midpoint temperatures (T_m_) with an approximately Gaussian distribution spanning the same temperature range covered by the first DSC transition. In agreement with DSC, T_m_ has a mean value of 324.1 ± 0.2 K. As expected, in agreement with the broad non-cooperative reversible transition, groups of protons with similar T_m_ do not localize in specific structural regions (Fig. 3A) thus defining the lack of hierarchical behavior in this transition. A three state unfolding mechanism was also ruled out, as it was not possible to fit the NMR data with such a model.

Interestingly, consistently with what observed in phosphate^15^, also in Tris some resonances still preserved a good spectral dispersion and small chemical shift perturbations at the highest measured temperatures (Fig. 3B); their mapping on Ros87 NMR structure indicate the existence at 343 K of a metal binding structural intermediate constituted by the β-hairpin containing the two coordinating cysteines, the last turn of the first helix containing the second coordinating histidine and part of the second helix.

As already mentioned above, the DSC thermogram shows a second reversible transition that conversely resulted irreversible in phosphate buffer and attributable to the final loss of the metal ion and of the intermediate residual structure.

The effect of a systematic change of ionic strength and pH conditions in Tris buffer on Ros87 unfolding was also examined via far-UV CD spectroscopy (Figure SI 4). In all the cases, it was not possible to fit the obtained data to either a two- or three-state model. The melting profile obtained appear quite similar to the profile reported in Fig. 2, strongly indicating that Ros87 unfolding is certainly downhill over a wider range of conditions.

### Apo-Ros87 and Ros87_C27D thermal unfolding

**Figure 4:**
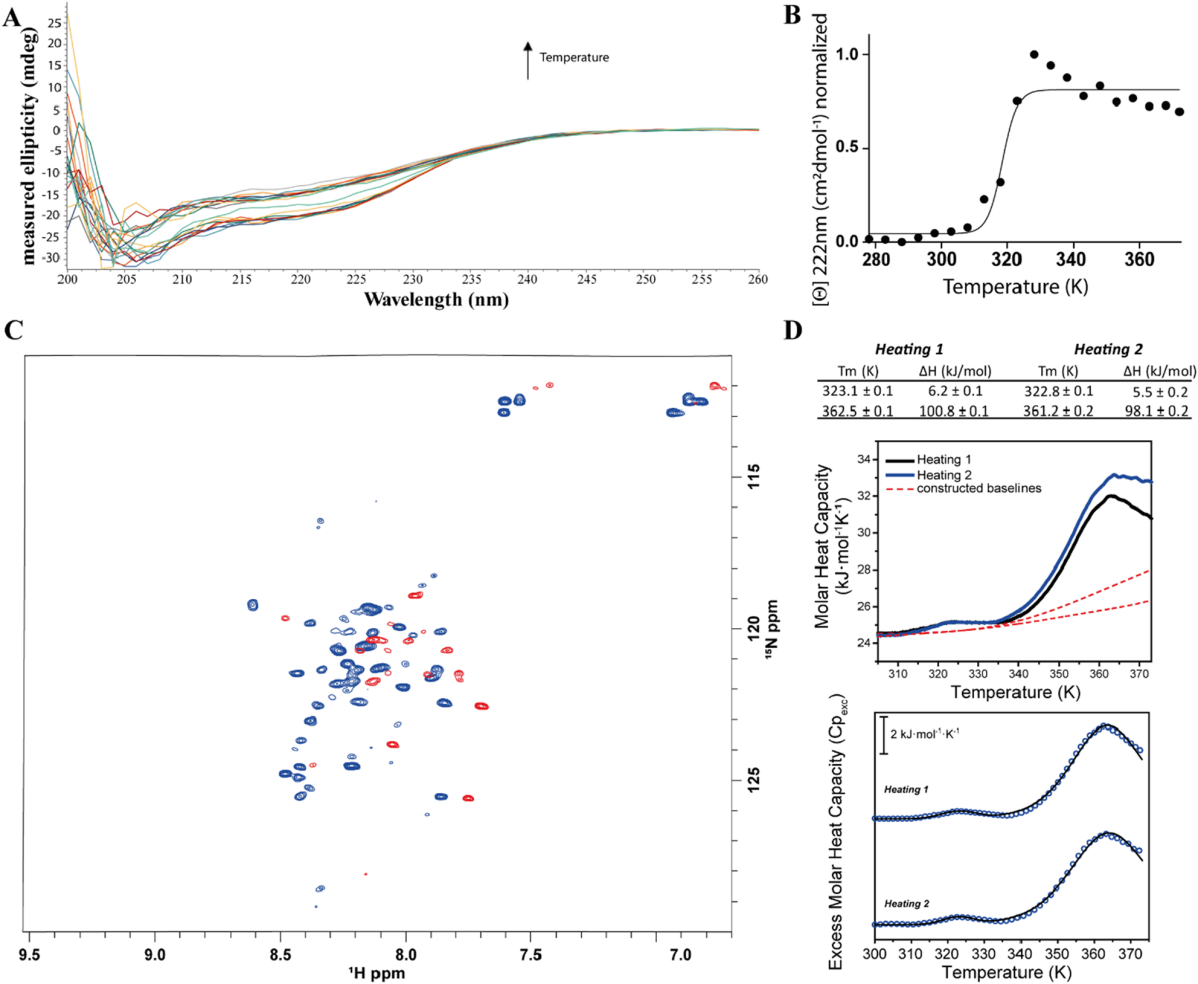
*Apo-Ros87 thermal unfolding.* (**A**) CD spectra recorded at increasing temperatures (298–372 K). (**B**) CD melting followed at 222 nm in the temperature range 298–372 K. (**C**) Overlay of the ^1^H–^15^N HSQC spectra of apo-Ros87 at 298 K (blue) and at 318 K (red). (**D**) DSC thermal unfolding curves and fitting of two different heating cycles of apo-Ros87.

In order to investigate the second transition of the DSC thermogram, we have decided to explore the thermal denaturation behavior of the apo-protein. We have previously shown that the structural metal ion removal causes severe effects on Ros87 structure and stability^41^: apo-Ros87 misses tertiary structure interactions and adopts flexible conformations that lack ordered secondary structures as well (Figure SI 5A). However, some slight differences with the complete random coil protein were also evidenced. Therefore, we have decided to investigate its thermal behavior via CD, DSC and NMR. The far-UV CD spectrum of apo-Ros87 was characteristic of an unfolded polypeptide with reduced amount of secondary structure content when compared with that of the metal loaded protein (Figure SI 5B). Yet, the depth of the spectrum around the 222 nm wavelength suggests a residual helical structure content. This could result from the formation of transient α-helices at different sites of the unfolded protein and/or from the presence of a small local core that resists the destabilizing effects of metal removal. In agreement with its largely unfolded state, the protein does not exhibit a cooperative unfolding transition when the CD signal at 222 nm was monitored while raising temperature (Fig. 4A), but again the presence of residual structure is suggested by the decrease in amplitude of the 222 nm signal at 316.4 ± 1.8 K (Fig. 4B and SI 5C).

NMR data appear in substantial agreement with the CD analysis. In fact, in contrast to Ros87, ^1^H–^15^N HSQC spectrum of the apo-form (Figure SI 5A) is poorly dispersed in both proton and nitrogen dimensions and lacks any subset of well dispersed signals indicating that the loss of structure is severe and involves all protein regions and that the residual secondary structure shown by the CD spectrum is not due to a core region that remains folded in spite of the destabilizing effect of the metal lacks. Accordingly, the temperature increase results in a change of the distribution pattern of the ^1^H–^15^N HSQC resonances above 318 K (Fig. 4C and SI 6). DSC data (Fig. 4D) reconcile with CD and NMR data showings a first small reversible transition centered at 323.1 (± 0.1)K. Interestingly apo-Ros87 DSC thermogram shows, similarly to Ros87, a second broad reversible transition centered at 364.1 (± 0.2)K. We fitted the DSC profile of this second transition according to the variable-barrier model (Figure SI 7). As expected, the fitted energy barrier value β was very high (30 kJ/mol^−1^) thus confirming that this unfolding transition cannot be considered downhill. This transition fitted perfectly to a two-state model with a 1.010 (± 0.005) van’t Hoff to calorimetric enthalpy ratio (ΔH_vH_∕ΔH_cal_).

**Figure 5:**
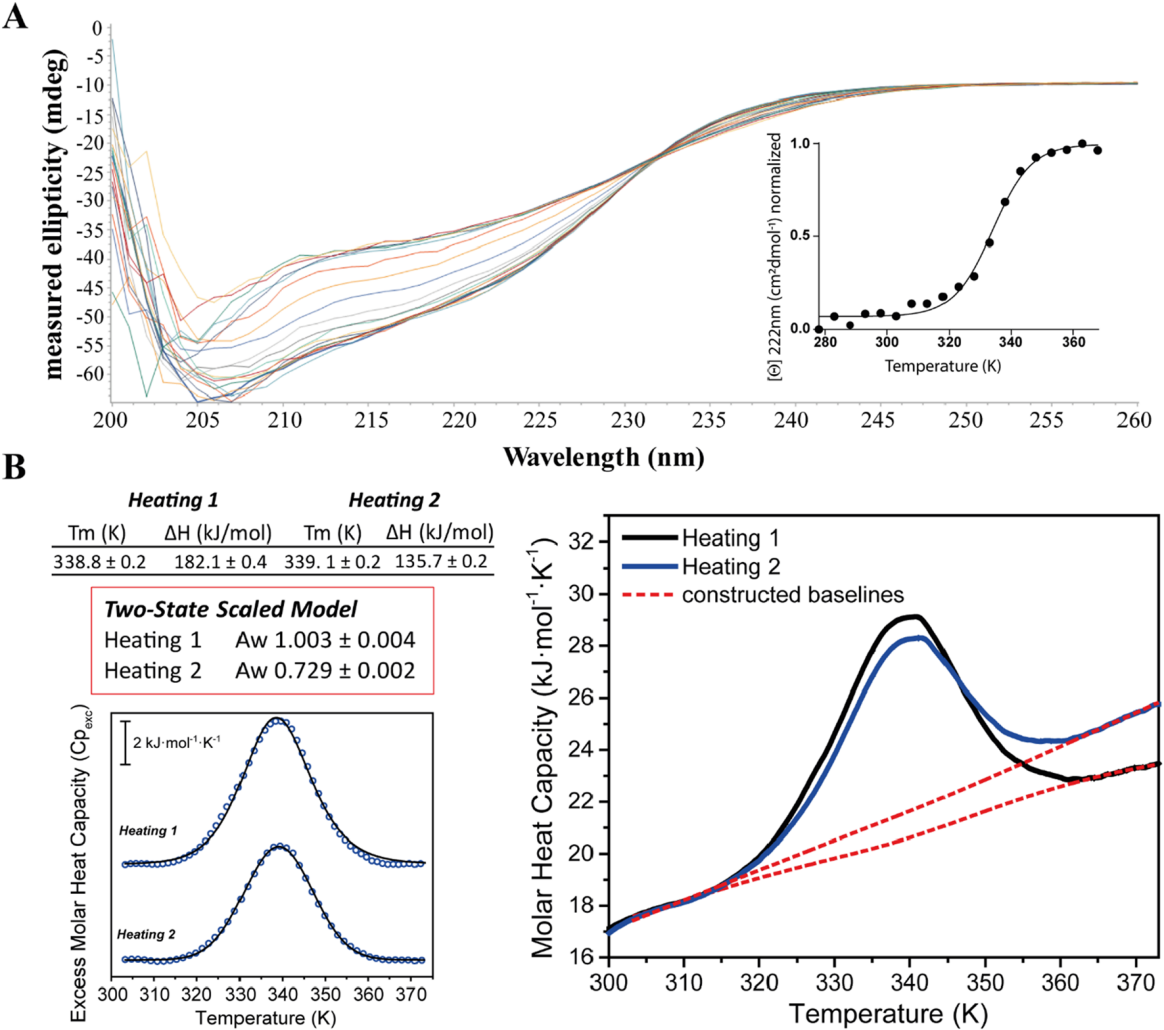
*Ros87_C27D thermal unfolding.* (**A**) CD thermal unfolding. Data were fitted to a two state model. (**B**) DSC thermal unfolding curves and fitting of two different heating cycles of Ros87_C27D.

In order to understand the conformational determinants governing this second transition we investigated also the thermal behaviour of Ros87_C27D, a single point mutant of Ros87 in which the second coordinating cysteine is mutated in aspartate^42,43^. This mutant has been previously shown to fold around the zinc ion and to overall preserve Ros87 global fold and positions of the secondary structure elements^42^. Ros87_C27D shows only minimal structural alterations around the mutation site and for few hydrophobic residues, revealing a slight local reorganization of the protein core. However, these small structural changes resulted in a clear change in the unfolding mechanism observed for this mutant via far-UV CD: unlike the wild-type protein this mutant faces a two-state folding mechanism (Fig. 5A).

Accordingly, the DSC analysis here reported shows for this mutant a behavior strikingly different from that of the wild-type protein. In particular, the DSC thermogram shows a unique reversible transition centered at 338.8 ± 0.2 K accompanied by an enthalpic change of about 182 kJ/mol in the first heating cycle and 136 kJ/mol in the second heating cycle. Cp_exc_(T) traces (Fig. 5B) fit perfectly to a two-state model with an average ΔH_vH_∕ΔH_cal_ of 1.003 ± 0.04, supporting the previous mentioned findings which indicate the lack of intermediate species during the transition. DSC data fitted to the variable-barrier model are reported in Figure SI 8. The fitted energy barrier value β was very high (25 kJ/mol^−1^) also in this case, thus confirming that Ros87_C27D unfolding cannot be considered downhill.

Interestingly, Ros87_C27D thermogram does not show the second transition seen for the wild type protein. Overall, data collected on apo-Ros87 and on Ros87_C27D allow us to conclude that the second transition seen for apo-Ros87 and Ros87 is likely due to the loss of metal coordination (in Ros87) and the concurrent transition to partially insoluble aggregated forms^44^ involving the two unbound cysteines.

## Discussion

The body of data collected in the present work clearly shows that change of buffer, pH and ionic strength does not influence Ros87partly downhill unfolding mechanism. Differences with what was previously seen in phosphate buffer are found in the second transition and are likely due to the different environment found by the cysteines during metal ion release. This however does not influence the folding/unfolding behavior of the protein described by the first DSC transition indicating that the rate-limiting step in Ros87 folding is represented by zinc recruitment and, once metal ion is recruited, a partially folded intermediate forms and the protein moves toward its native functional state with a non-cooperative downhill mechanism.

Downhill folding mechanisms were originally supposed to occur only for proteins with particularly optimized native interactions, under strongly stabilizing conditions^35,45^ or when favorable mutations occurred^36^. We aim at contributing to the general discussion indicating that, at least in metallo-proteins, downhill folding can be commonly found under a much wider range of conditions and coupled to other different transitions. The key event in determining the mechanism of folding is the organization of metal ion center that not only stabilizes the native state but is determinant in the folding process of this proteins family.

The finding that mutations of Ros87 zinc coordination sphere trigger different folding mechanisms resembling a more common two-state process suggests that the complex two-step folding mechanism found in Ros87, including a stable downhill scenario, is a precise evolutionary result. The biological advantage of evolving such bipartite folding process needs further investigations but it could have been driven by the necessity to recruit and retain the metal ion in the most diverse chemical conditions.

## Methods

### Protein expression and purification

Un-labelled and ^15^N labelled proteins used for CD, NMR and DSC experiments were over-expressed and purified as formerly reported^22^. Concisely, transformed *E. coli* host strain BL21(DE3) were plated onto an LB-agar plate containing ampicillin (100 µg/ml) for selection. For ^15^N labeling, cells were grown at 37° in a minimal medium that contained 0.5 g/l ^15^NH_4_Cl as only nitrogen source. At O.D._600_ ~ 0.6 the expression was induced with 1.0 mM isopropyl-β-d-thiogalactopyranoside (IPTG) for 1 h 30 min. Cells were harvested by centrifugation (3750 rpm for 40 min) and the pellet was re-suspended in 20 mM Na_2_HPO_4_ (pH 6.8) buffer. The suspension was lysed using a sonicator and centrifuged at 16,500 rpm for 40 min. The supernatant was filtered through a 0.22 µm filter membrane to remove cell debris and applied to a Mono S HR 5/5 cation exchange chromatography column (Amersham Bioscences) equilibrated with phosphate buffer. The fractions that contained the proteins were applied to a HiLoad 26/60 Superdex 75 (Amersham Bioscences) gel filtration chromatography column equilibrated with 20 mM Na_2_HPO_4_ (pH 6.8), 0.2 M NaCl. For CD and UV–Vis experiments, unlabeled proteins were expressed in LB-medium and the purification procedure described above was followed. An Amicon ultra-15 centrifugal filter was used to concentrate the proteins after the purification phase to reach the desired final concentration. The zinc ion was removed from obtained samples by acidifying Zn(II)-Ros87 at pH ~ 2.5 adding HCl 0.1 M and dialyzing it against 10 mM Tris, 150 µM TCEP (400 µM for NMR samples) aqueous solution at pH ~ 3.0. The pH was fixed to 6.5 and controlled throughout the experiments. Tris buffer and TCEP were chosen to avoid precipitates formation and to prevent cysteine oxidation. Both Tris and TCEP experience a weak affinity for zinc that does not interfere with the measurements^46,47^.

### CD spectroscopy

Ros87 and apo-Ros87 in 10 mM Tris, 150 µM TCEP, at pH 6.5 were thermally denaturated by using a JASCO J-815 CD spectropolarimeter equipped with Peltier temperature control. Experiments were repeated in the same buffer also at different pH values (6, 7.5 and 8.5) and at different ionic strengths (100, 300 and 400 mM NaCl). CD spectra were measured at 5 K intervals in the temperature range of 278–373 K (3 K for Zn(II)-Ros87). At the end, samples were cooled back to 298 K and a final set of spectra recorded. Data were collected using a quartz cuvette with a 1 cm path-length in the 200–260 nm wavelength range with 1 nm data pitch and 50 nm/min scanning speed. Data, normalized against reference spectra to remove buffer background contribution, were fitted by two-state or three-state model.

### NMR spectroscopy

NMR samples were made of ~ 250 μM protein solution, 10 mM Tris, 400 μM TCEP adjusted to pH 6.5 in 550 μL of 90% H_2_O/10% ^2^H_2_O. The NMR spectra were recorded on a Bruker Avance III HD 600 MHz, equipped with a cryoprobe, at the Department of Environmental, Biological and Pharmaceutical Sciences and Technologies, University of Campania “L. Vanvitelli” (Caserta, Italy). A series of ^1^H–^15^N HSQC spectra were acquired increasing temperatures at regular intervals of 5 K from 298 to 343 K with: 256 complex points for ^15^N (F1), 1024 for ^1^H (F2). The ^1^H and ^15^N chemical shifts were indirectly calibrated using Tetramethylsilane as external reference. Data were processed using TopSpin 3.5 (Bruker) and NMRPIPE^48^, analyzed with CARA^49^ and SPARKY^50^ software. The structures were visualized and evaluated using the software CHIMERA^51^ and PyMol^52^.

### Differential scanning calorimetry (DSC)

Protein samples were prepared after extensive dialysis against the buffer (10 mM Tris, 150 µM TCEP, pH 6.5). Next, after vacuum degassing, they were heated at 1 K/min scan rate in the temperature range 278–373 K. A 3 atm extra external nitrogen pressure was applied to samples to prevent formation of air bubbles during heating. In all measurements, the reference cell of the calorimeters was filled by the buffer from the last dialysis step. To ensure an accurate equilibration of the calorimeter, several buffer–buffer heating scans were routinely performed before DSC measurement. To obtain the molar heat capacity curves Cp(T), buffer–buffer/metal baselines were recorded at the same scanning rate, subtracted from raw DSC curves and normalized by the protein concentration. Excess molar heat capacities curves (Cp_exc_) were obtained from Cp(T), by subtracting a baseline obtained by a fifth-order polynomial fit of pre- and post-transition Cp trends as described elsewhere^53^. As previously observed for other metallo-proteins^54,55^, the heat capacity curve of the unfolded protein is significantly distorted due to the concomitant occurrence of phenomena linked to the presence of metal ions. For this reason, we estimated the baseline by using a fifth-order polynomial fit. For all experiments, two consecutive heating–cooling cycles were performed to determine the process reversibility. This value was calculated as the enthalpy change ratio of the second scan (reheating) vs. the first one. Cp_exc_ curves were deconvoluted by NanoAnalyze software using the Gaussians model. The number of DSC components to be adopted in peak deconvolution procedure was selected in order to minimize fitting errors. DSC experiments were run by a NanoDSC instrument (TA Instruments).

## Supplementary information

Supplementary Information.
