# Supplementary material for: The change of conditions does not affect Ros87 downhill folding mechanism
Source: Sci Rep. 2020 Dec 3;10:21067. doi: 10.1038/s41598-020-78008-8 (PMC7713307; doi:10.1038/s41598-020-78008-8)
Supplement: Supplementary file 1 — Supplementary Information. [file 41598_2020_78008_MOESM1_ESM.docx]

**The change of conditions does not affect Ros87 downhill folding mechanism**

Rinaldo Grazioso^1^, Sara García-Viñuales^2^, Gianluca D’Abrosca^1^, Ilaria Baglivo^1^, Paolo Vincenzo Pedone, Danilo Milardi^2^, Roberto Fattorusso^1^, Carla Isernia^1^, Luigi Russo^1*^, Gaetano Malgieri^1*^

1: Department of Environmental, Biological and Pharmaceutical Science and Technology, via Vivaldi 43, 81100 Caserta (Italy).

2: Institute of Crystallography-CNR, Via Paolo Gaifami 18, 95126 Catania (Italy).

* correspondence to: luigi.russo2@unicampania.it and gaetano.malgieri@unicampania.it

**Supplementary Information**

**
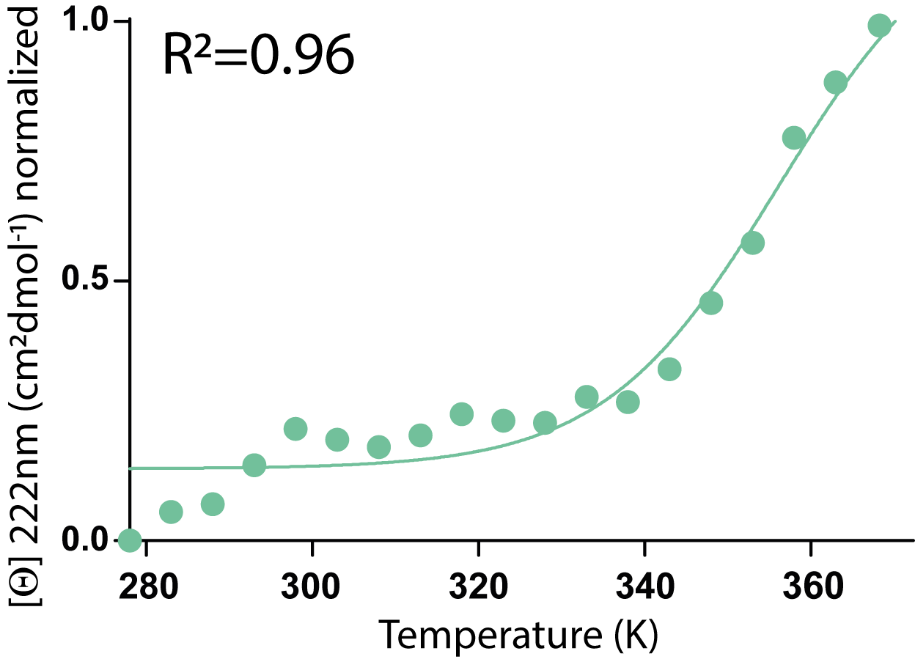
**

**Figure SI 1:** CD thermal unfolding of Ros87 - the line represents the fitting to a two-state folding model. The R^2^ value is indicative of a bad quality of the reported fitting.

**
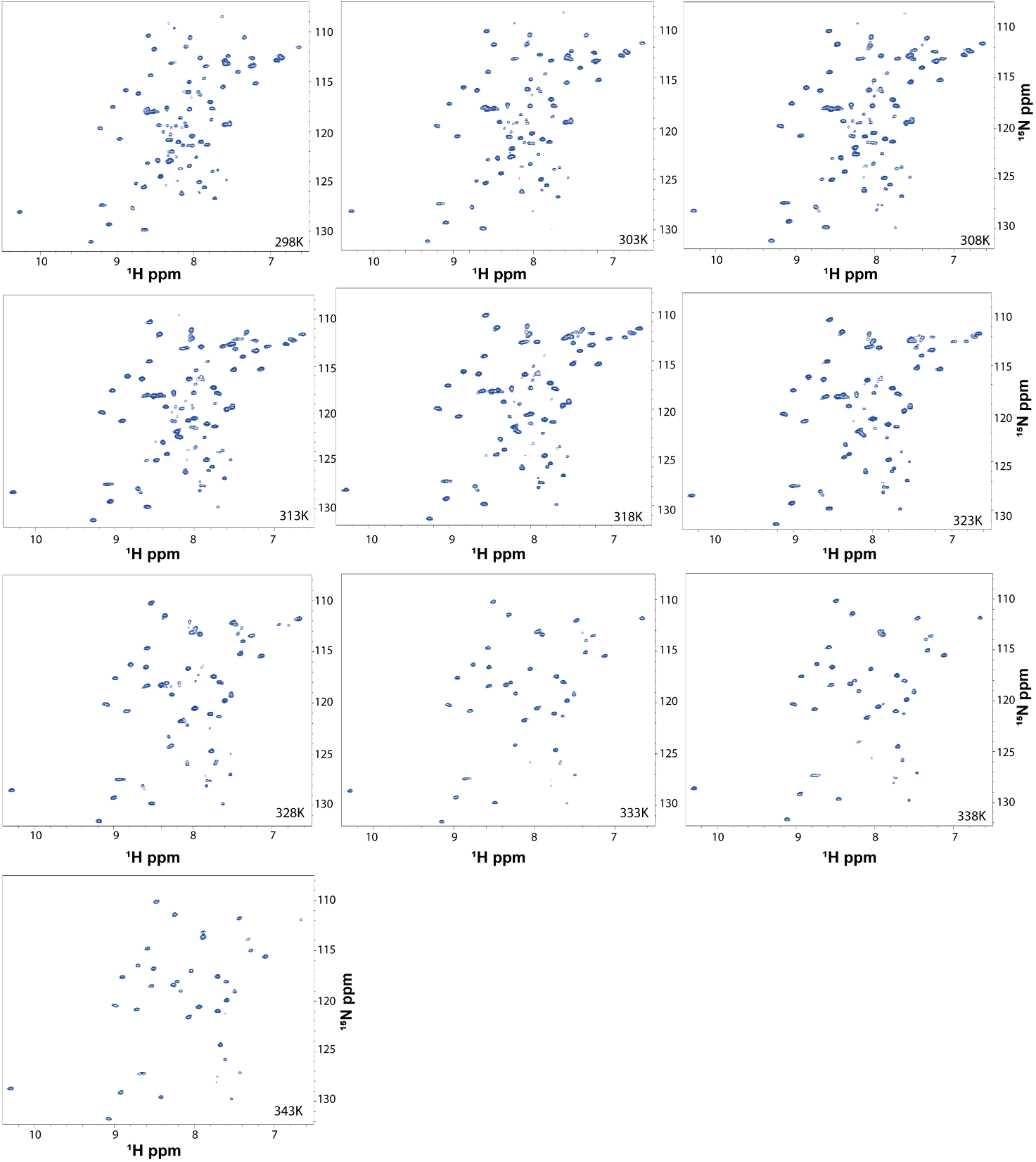
**

**Figure SI 2.** NMR thermal unfolding of Ros87: ^1^H-^15^N HSQC spectra at different temperatures (298-343 K).

**
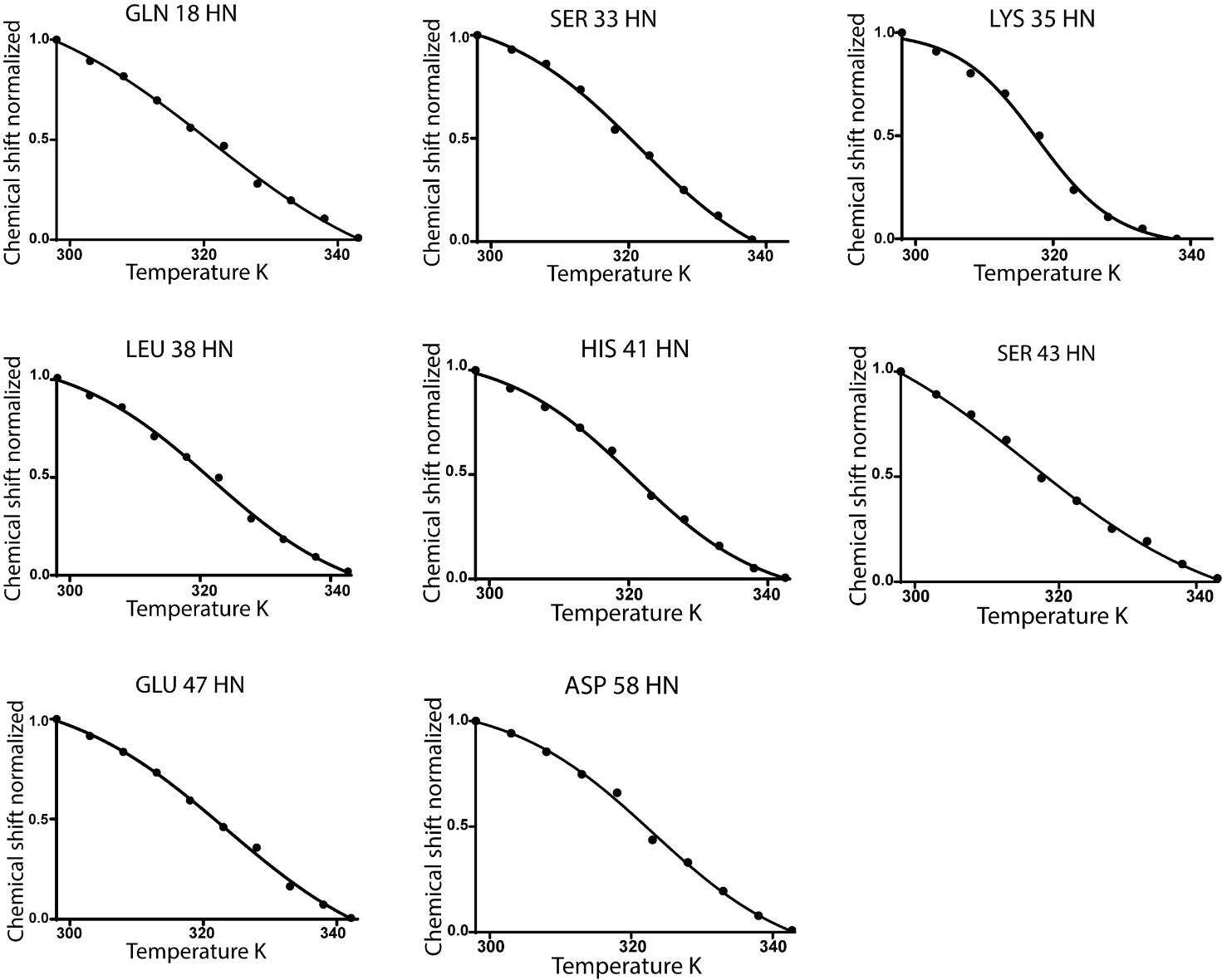
**

**Figure SI 3.** Representative HN thermal unfolding curves of Ros87. Data were fitted to a two-state model.


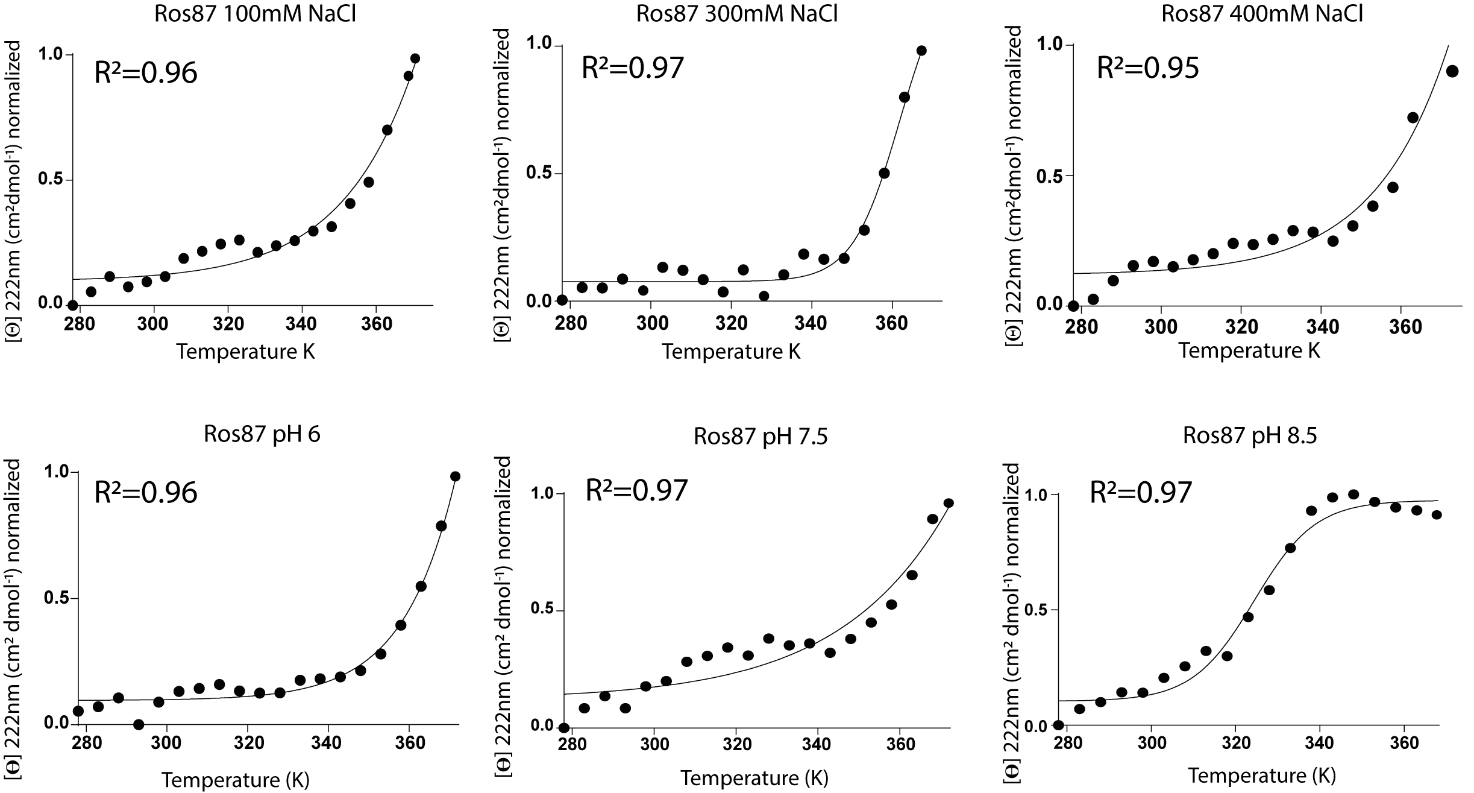


**Figure SI 4.** CD thermal unfolding of Ros87 in 10 mM Tris, 150µM TCEP. Each experiment was conducted at different conditions of pH or ionic strength. The lines represent the fitting to a two-state

folding model. The R^2^ value is indicative of a bad quality of the reported fitting.

.**
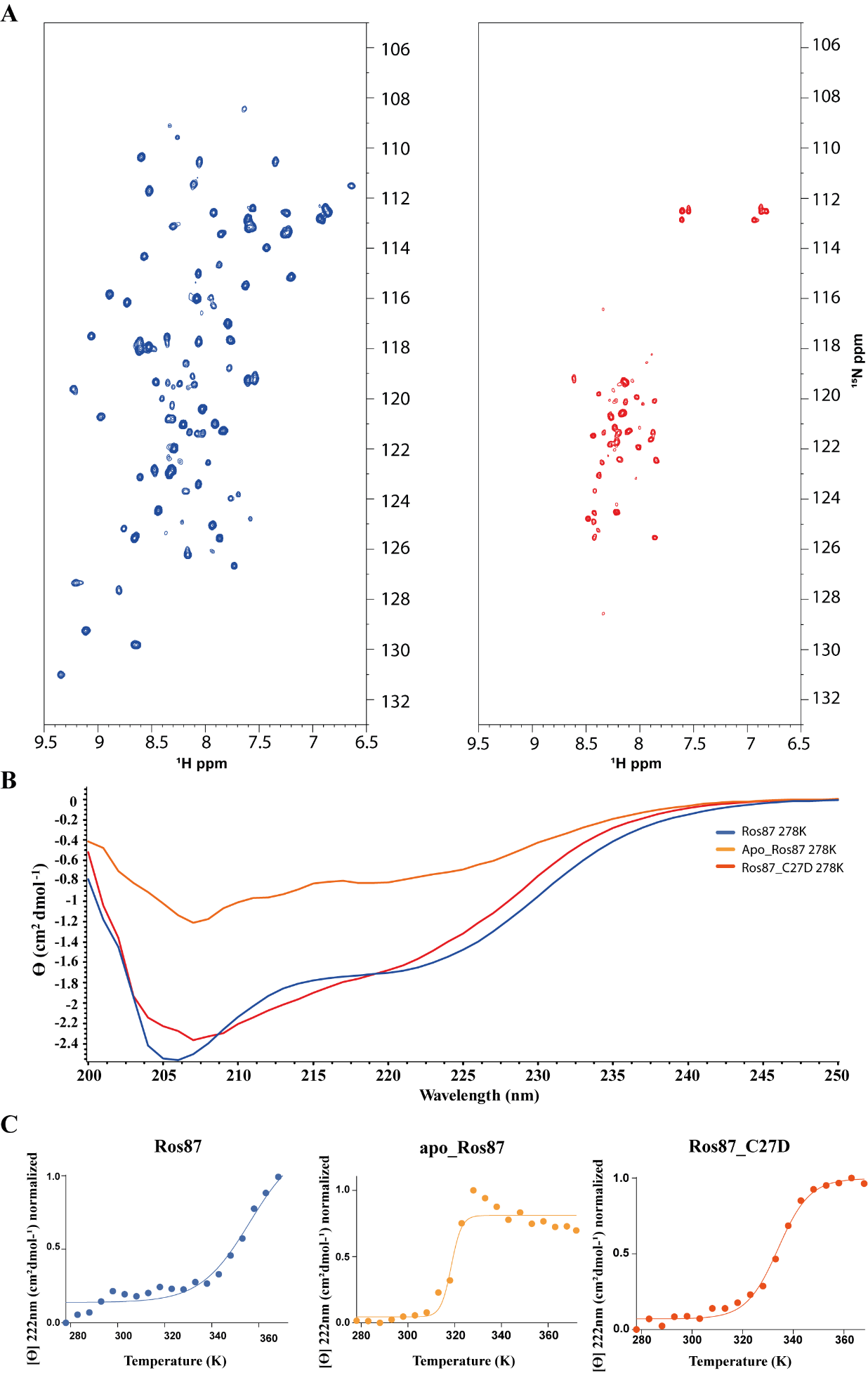
**

**Figure SI 5. (A)** Portion of the ^1^H-^15^N HSQC spectra at 278K of Ros87 (in blue) and apo-Ros87 (in red). **(B)** Overlay of Ros87, Ros87_C27D and apo-Ros87 CD spectra acquired at the lowest temperature investigated (278 K). **(C)** CD thermal unfolding of Ros87 (in blue), apo-Ros87 (in orange) and Ros87_C27D (in red). The lines represent the fitting to a two-state folding model.


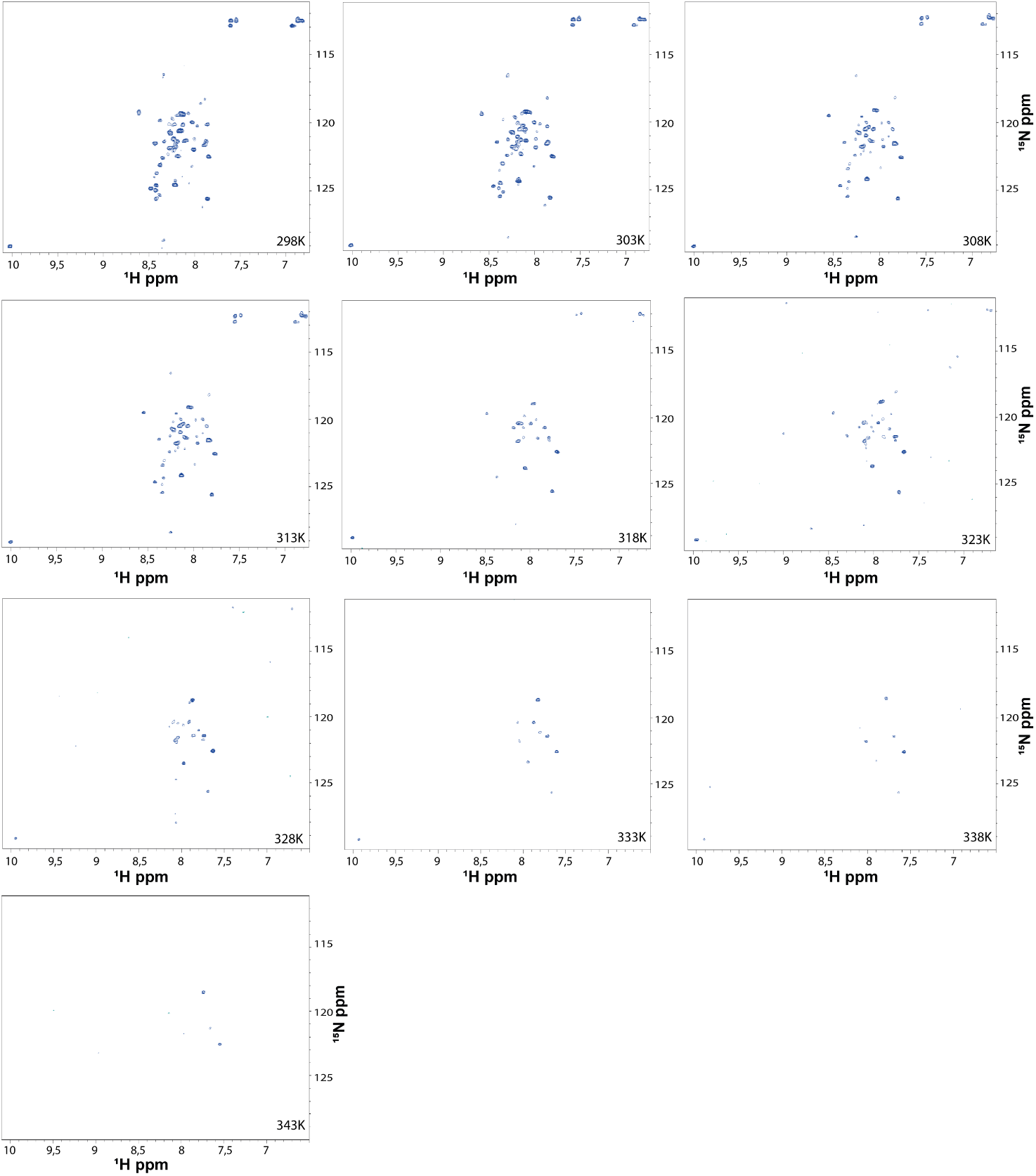


**Figure SI 6.** NMR thermal unfolding of Apo-Ros87: ^1^H-^15^N HSQC spectra at different temperatures (298-343 K).

**
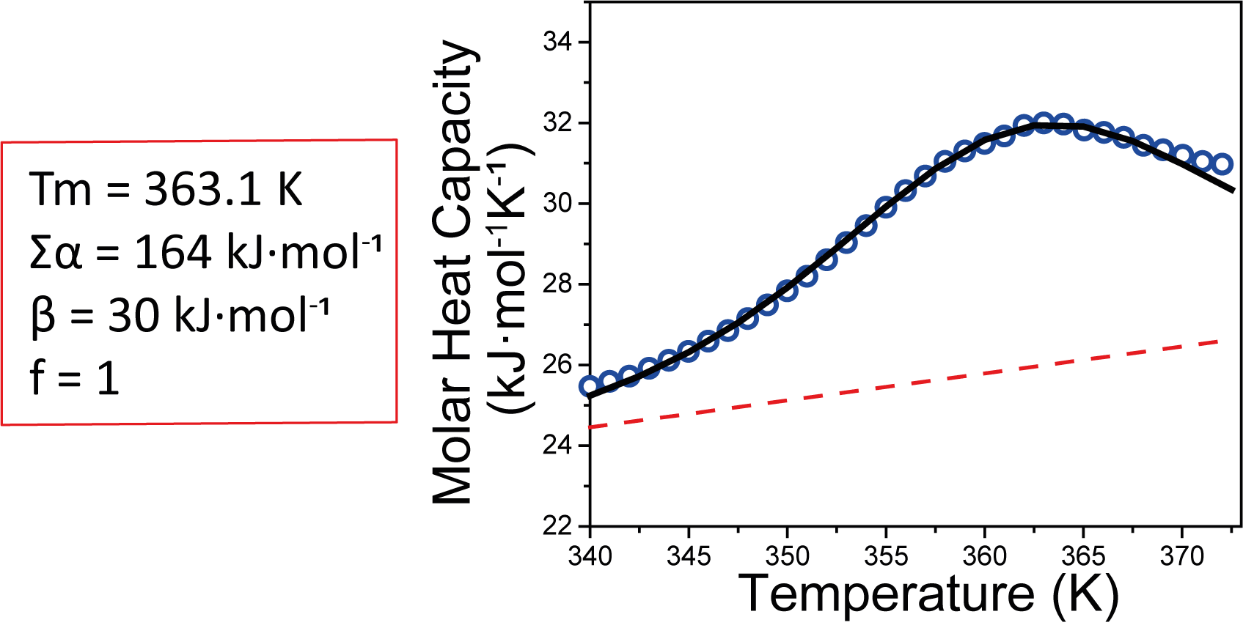
**

**Figure SI 7.** DSC thermal unfolding and fitting of Apo-Ros87. Data were fitted to a downhill folding model. The constructed baseline is reported as a red dashed line.


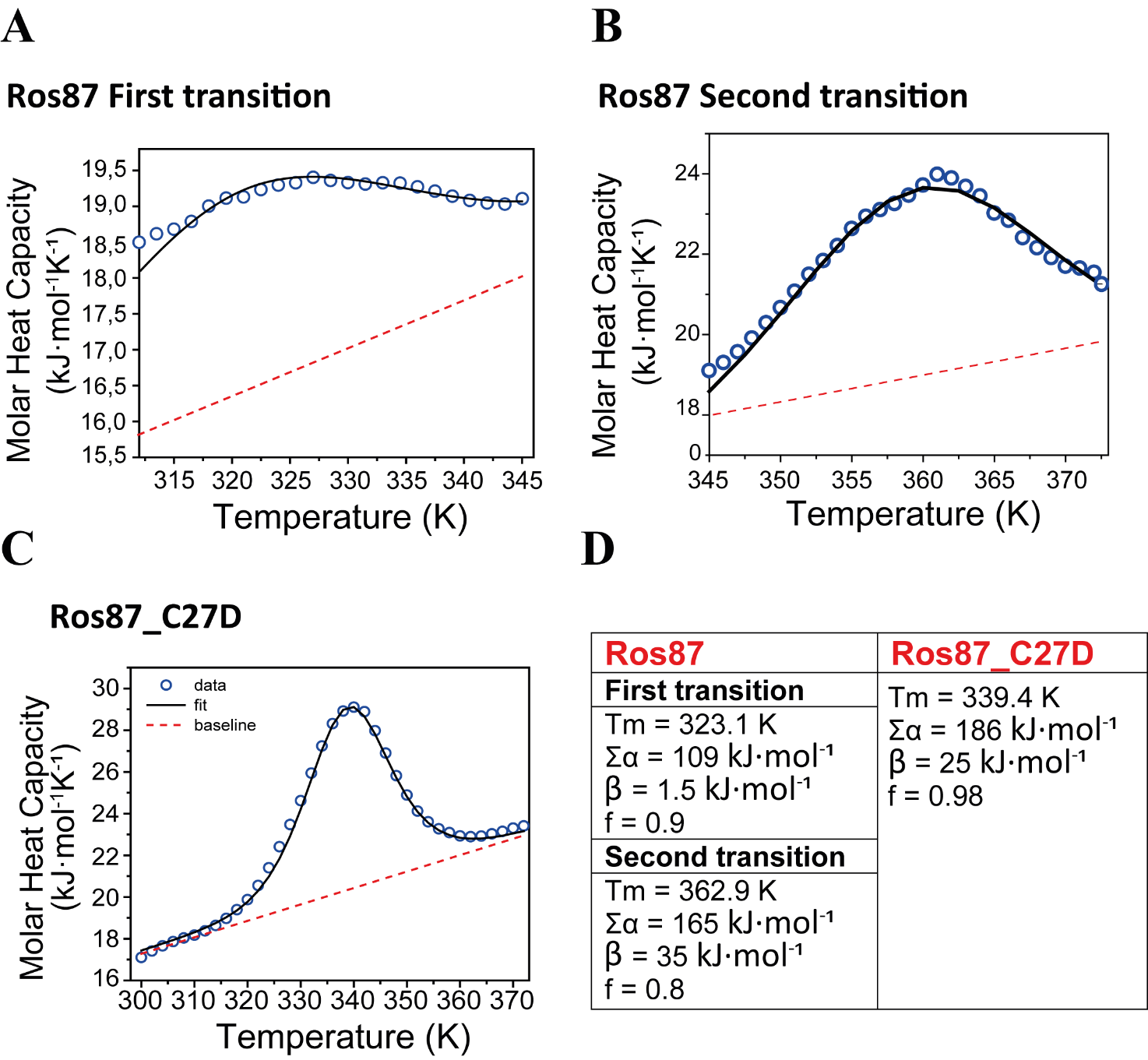


**Figure SI 8.** DSC thermal unfolding and fitting of Ros87 and Ros87_C27D. Data were fitted to a downhill folding model. The constructed baselines are reported as a red dashed line.
